# Role of circulating inflammatory protein in the development of diabetic renal complications: proteome-wide Mendelian randomization and colocalization analyses

**DOI:** 10.3389/fendo.2024.1406442

**Published:** 2024-07-08

**Authors:** Wenli Liu, Jiaqi Zhang, Duo Zhang, Lei Zhang

**Affiliations:** ^1^ State Key Laboratory of Dampness Syndrome of Chinese Medicine, The Second Clinical Medical College of Guangzhou University of Chinese Medicine, Guangzhou, China; ^2^ Department of Nephrology, The Second Affiliated Hospital of Guangzhou University of Chinese Medicine, Guangzhou, China; ^3^ Guangdong Provincial Key Laboratory of Clinical Research on Traditional Chinese Medicine Syndrome, The Second Affiliated Hospital of Guangzhou University of Chinese Medicine, Guangzhou, China

**Keywords:** diabetic with renal complications, circulating inflammatory proteins, Mendelian randomization, colocalization, causality, risk

## Abstract

**Background:**

Diabetes ranks among the most widespread diseases globally, with the kidneys being particularly susceptible to its vascular complications. The identification of proteins for pathogenesis and novel drug targets remains imperative. This study aims to investigate roles of circulating inflammatory proteins in diabetic renal complications.

**Methods:**

Data on the proteins were derived from a genome-wide protein quantitative trait locus (pQTL) study, while data on diabetic renal complications came from the FinnGen study. In this study, proteome-wide Mendelian randomization (MR) and colocalization analyses were used to assess the relationship between circulating inflammatory proteins and diabetic renal complications.

**Results:**

MR approach indicated that elevated levels of interleukin 12B (IL-12B) (OR 1.691, 95%CI 1.179–2.427, P=4.34×10^-3^) and LIF interleukin 6 family cytokine (LIF) (OR 1.349, 95%CI 1.010–1.801, P=4.23×10^-2^) increased the risk of type 1 diabetes (T1D) with renal complications, while higher levels of fibroblast growth factor 19 (FGF19) (OR 1.202, 95%CI 1.009–1.432, P=3.93×10^-2^), fibroblast growth factor 23 (FGF23) (OR 1.379, 95%CI 1.035–1.837, P=2.82×10^-2^), C-C motif chemokine ligand 7 (CCL7) (OR 1.385, 95%CI 1.111–1.725, P=3.76×10^-3^), and TNF superfamily member 14 (TNFSF14) (OR 1.244, 95%CI 1.066–1.451, P=5.63×10^-3^) indicated potential risk factors for type 2 diabetes (T2D) with renal complications. Colocalization analysis supported these findings, revealing that most identified proteins, except for DNER, likely share causal variants with diabetic renal complications.

**Conclusion:**

Our study established associations between specific circulating inflammatory proteins and the risk of diabetic renal complications, suggesting these proteins as targets for further investigation into the pathogenesis and potential therapeutic interventions for T1D and T2D with renal complications.

## Introduction

Diabetes remains a significant global health concern, with approximately 463 million cases reported worldwide in 2019 (1). The kidneys are particularly susceptible organs to diabetic vascular complications, leading to glomerular disorders primarily manifested as diabetic nephropathy (DN) (2). DN has emerged as the principal cause of end-stage renal disease (ESRD) (3). DN is characterized by proteinuria and a sustained decline in the estimated glomerular filtration rate (eGFR), with its pathogenesis likely involving oxidative stress, hemodynamic imbalances, chronic inflammation, and fibrosis (4, 5). Most importantly, an increasing number of studies have considered that chronic inflammation is a key driver of diabetes complications, especially in the development of DN (6–8). It was demonstrated that immune cells (such as lymphocytes, macrophages, and neutrophils) were involved in the emergence and development of DN, and that maintaining the immune homeostasis of these cells reduced the production of pro-inflammatory cytokines, such as interleukin 1β (IL-1β), IL-6, tumor necrosis factor alpha-like (TNF-α), and monocyte chemoattractant protein 1(MCP-1), thereby ameliorating the progression of DN (9–12). Assessing the role of inflammatory proteome in diabetic renal complications is crucial to elucidate their pathophysiology and explore potential therapeutic targets.

Plasma proteins play an essential role in a range of biological processes, such as signaling and modulation of inflammation (13). The imbalance between pro-inflammatory and anti-inflammatory immunoregulatory responses, driven by inflammatory proteins, is hypothesized to contribute to DN pathogenesis, including proteinuria, extracellular matrix (ECM) accumulation, and a progressive decrease in eGFR (14). Genome-wide association studies (GWAS) have identified genetic variants that influence circulating inflammatory protein concentrations, represented by strongly correlated single nucleotide polymorphisms (SNPs) and known as protein quantitative trait loci (pQTL) (15). These findings provide significant materials to examine the causal effects of inflammatory proteins on diabetic renal complications through Mendelian randomization (MR) analysis.

MR analysis leverages genetic variations from GWAS summary statistics as instrumental variables (IVs) to discern causal links between exposures and outcomes, circumventing confounding biases and reverse causation inherent in observational studies (15). Given the random categorization of genetic variants during meiosis akin to the procedure in a randomized controlled trial, and the lifetime impact of genetic variation, MR analysis is preferable for detecting the long-term causal effects of risk or protective factors on outcomes (16). For a genetic instrument to be deemed valid, it must satisfy three critical assumptions: (i) It exhibits causality related to the exposure; (ii) it remains uninfluenced by confounding variables; (iii) its association with the outcome is mediated solely through the exposure. Based on publicly available GWAS data, we conducted a proteome-wide MR analysis to investigate the causal relationships between circulating inflammatory proteins and diabetic renal complications. In addition, we performed colocalization analysis in order to assess whether the identified proteins and diabetic renal complications existed shared causal variants.

## Methods

### Study design and data sources

**Figure 1:**
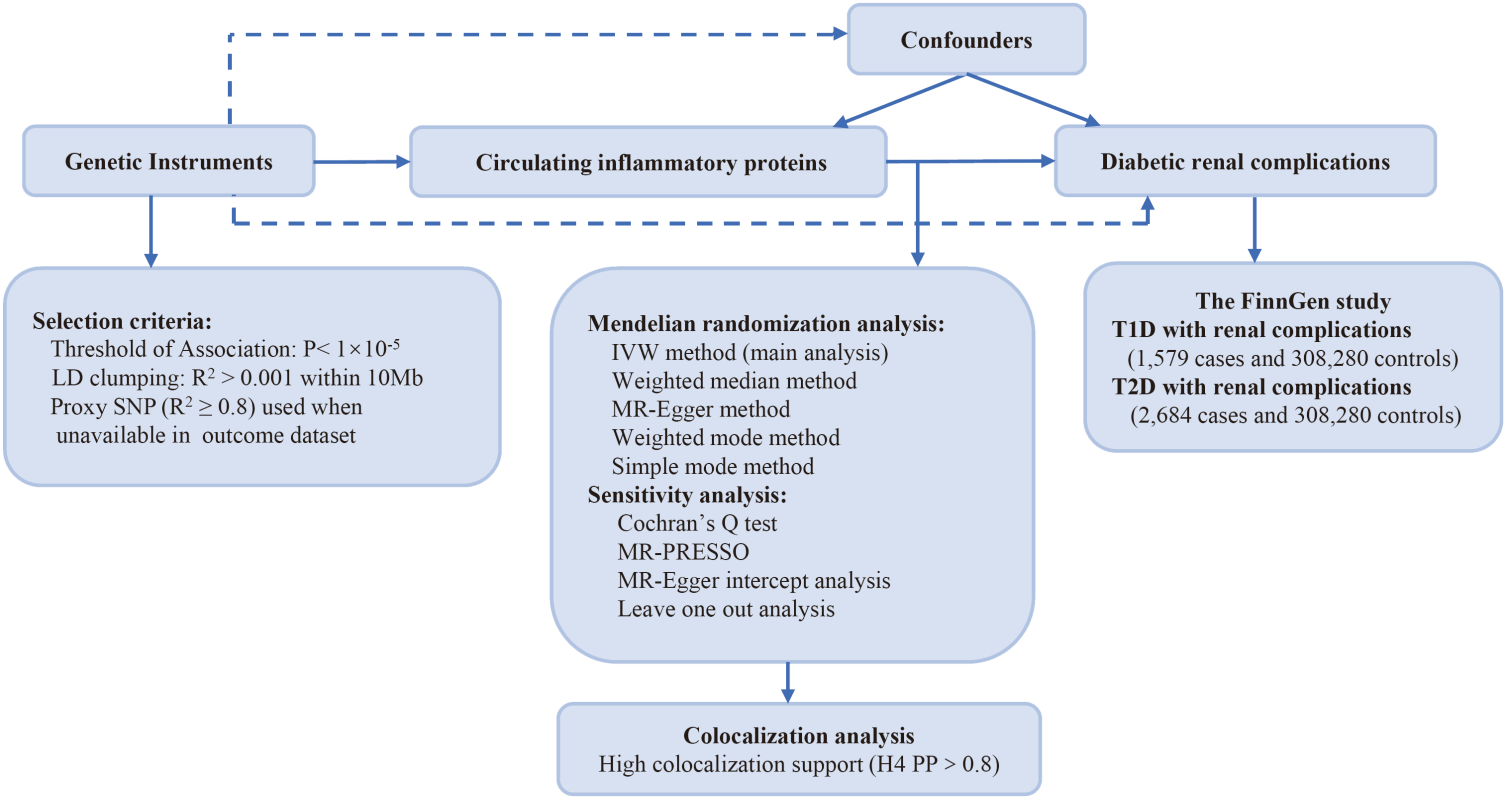
Study design: investigating causal relationships between 91 circulating inflammatory proteins and diabetic renal complications. LD, linkage disequilibrium; SNP, single nucleotide polymorphism; IVW, inverse-variance weighted; PP: posterior probability; T1D, type 1 diabetes; T2D, type 2 diabetes.

**Figure 1** illustrates the research design employed in this investigation. We explored the causal links between genetically predicted levels of circulating inflammatory proteins and diabetic renal complications through MR analysis. Genetic instruments pertaining to circulating inflammatory proteins were derived from a genome-wide pQTL study within the SCALLOP Consortium’s framework. The study utilized the Olink Target platform to measure 91 circulating inflammatory proteins across 11 cohorts, totaling 14,824 individuals of European descent (17). The summary statistics of type 1 diabetes (T1D) (1,579 cases and 308,280controls) and type 2 diabetes (T2D) (2,684 cases and 308,280 controls) with renal complications were extracted from the FinnGen study R9 (18). Patients were diagnosed with type 1 diabetes with renal complications (E10.2†) or type 2 diabetes with renal complications (E11.2†), in accordance with the International Classification of Diseases, 10th Edition (ICD-10). In the ICD-10, diabetes with kidney complications is a subclassification, specifically referring to glomerular disorders in diabetes mellitus (N08.3*). All participants included in this analysis were of European ancestry, with no overlapping between the datasets for exposure and outcomes.

To ascertain relevant SNPs for each circulating inflammatory protein, the following methods were applied. As a limited number of SNPs met the genome-wide significant threshold (p<5×10^-8^) with circulating inflammatory proteins, we adopted a more lenient threshold of p<1×10^-5^ to select our IVs following the previous study (19). For multiple SNPs achieving genome-wide significance and exhibiting LD R^2^ > 0.001 within 10 Mb of loci, we selected the SNP with the lowest p-value linked to the traits of circulating inflammatory proteins. The SNPs referenced above were subsequently extracted from the GWAS on outcome traits. Based on the 1000 Genomes European reference panel, SNPs that are associated with proteins but are unavailable in the outcome data were replaced with SNP proxies that have a high linkage disequilibrium (R^2^ ≥ 0.8). We harmonized the exposure and outcome datasets and acquired the SNP effects along with their accompanying standard errors (20).

The GWAS summary statistics used in this study are publicly available, and all original research received ethical approval, thus the requirement for ethical approval of our study was waived.

### Statistical analysis

#### Mendelian randomization analysis

The inverse-variance weighted (IVW) method served as the principal analysis method, offering high-powered estimates contingent upon the postulate that all SNPs are valid IVs. The weighted median and MR-Egger methods refine the IVW-derived estimates by providing more robust assessments across a broader array of scenarios, but with reduced efficiency. The weighted median generates credible estimates when a minimum of 50% of the weight originates from effective genetic variants. If the effective genetic variance falls below 50%, MR-Egger can still generate pleiotropy robust estimates of causal effects. Furthermore, other approaches for two-sample MR, such as weighted mode and simple mode, were also used as references. Cochran’s Q test was conducted to assess the presence of heterogeneity. The MRPRESSO method was applied to detect and remove outliers potentially affected by horizontal pleiotropy. Additionally, the MR-Egger intercept and leave-one-out analyses were also undertaken to explore the potential pleiotropy (21). Noteworthy, associations with p-values below 2.75 × 10^-4^ (0.05/182, the Bonferroni correction) were deemed to be statistically significant evidence of association, whereas those in the range of 2.75 × 10^-4^ ~ 0.05 were considered suggestive of an association (22).

#### Colocalization analysis

To further investigate whether the identified proteins and diabetic renal complications existed shared causal variants, we conducted a colocalization analysis, which targeted inflammatory proteins significantly associated with diabetic renal complications according to MR results. We posited five distinct exclusion hypotheses for each genomic locus: H0 proposes no association with either trait; H1 suggests an association with protein levels but not the disease; H2 indicates an association with the disease but not protein levels; H3 implies associations with both traits but via distinct SNPs; and H4 posits a joint association with both traits through a common SNP. The analysis yields posterior probabilities for these hypotheses. Approximate Bayes factors were computed with the standard errors and effect estimates for each SNP, and the log Bayes factor was then computed for each hypothesis. Lastly, the posterior probability (PP) for each hypothesis was computed with the Bayes factor and the prior probability. Colocalization analysis was conducted on all variants that were within a 1 MB region (upstream or downstream) of the gene. We deemed the colocalization of an SNP with both traits as significant if the PP for H4 exceeded 80% (23).

All statistical analyses were performed using RStudio (version 4.2.2, Posit PBC, Boston, MA, USA) with the software packages “TwoSampleMR”, “MR-PRESSO” and “coloc R”.

## Results

### MR analysis

We evaluated the causal effects of genetically predicted levels of 91 circulating inflammatory proteins on T1D and T2D with renal complications. This study aimed to identify whether specific inflammatory proteins are causally linked to diabetes with renal complications and to pinpoint potential risk or protective factors, thereby informing future functional and clinical research. For example, detailed molecular mechanism studies on the identified proteins can elucidate their roles in the disease, advancing prevention and treatment strategies.

### Causal estimates of circulating inflammatory proteins on T1D with renal complications

**Figure 2:**
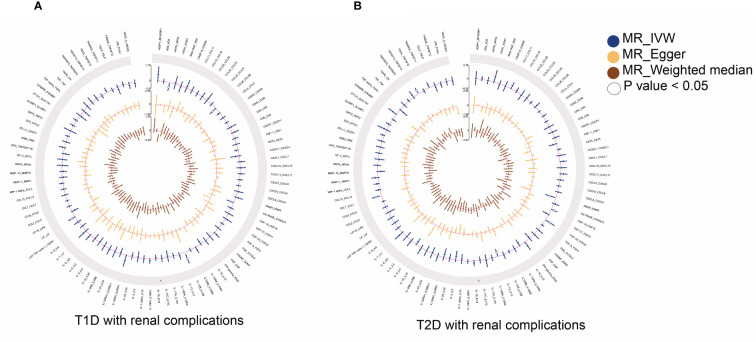
Summary results of 91 circulating inflammatory proteins and diabetic renal complications used IVW, MR-Egger, and weighted median methods in MR analysis. Circular plots show the MR results of 91 circulating inflammatory proteins in T1D **(A)** and T2D **(B)** with renal complications, respectively. MR, mendelian randomization; IVW, inverse-variance weighted; T1D, type 1 diabetes; T2D, type 2 diabetes.

**Figure 3:**
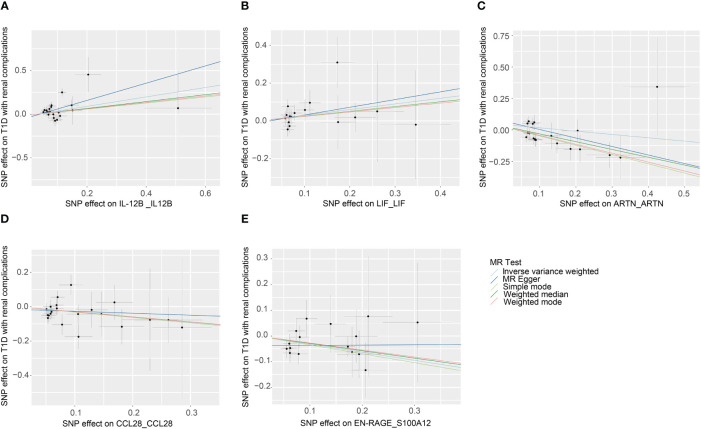
Scatter plots of causal associations for genetically predicted levels of IL-12B **(A)**, LIF **(B)**, ARTN **(C)**, CCL28 **(D)**, and S100A12 **(E)** on T1D with renal complications used IVW, MR-Egger, weighted median, weighted mode, and simple mode methods in MR analysis. MR, mendelian randomization; IVW, inverse-variance weighted; T1D, type 1 diabetes; IL-12B, interleukin 12B; LIF, LIF interleukin 6 family cytokine; ARTN, artemin; CCL28, C-C motif chemokine ligand 28; S100A12, S100 calcium binding protein A12.

**Figure 4:**
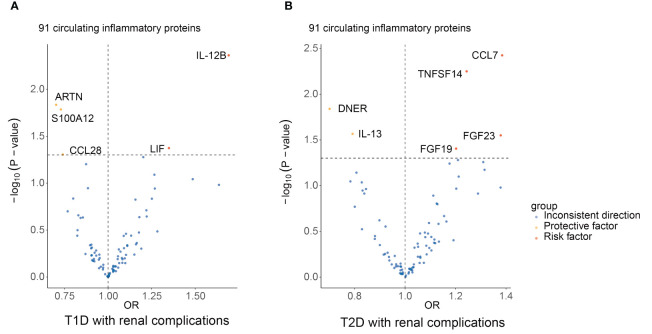
Summary results of 91 circulating inflammatory proteins and diabetic renal complications using the IVW method in MR analysis. Volcano plots show the MR results of 91 circulating inflammatory proteins on the risk of T1D **(A)** and T2D **(B)** with renal complications, respectively. MR, mendelian randomization; IVW, inverse-variance weighted; T1D, type 1 diabetes; T2D, type 2 diabetes; ARTN, artemin; CCL28, C-C motif chemokine ligand 28; S100A12, S100 calcium binding protein A12; IL12B, interleukin 12B; LIF, LIF interleukin 6 family cytokine; DNER, delta/notch like EGF repeat containing; IL-13, interleukin 13; FGF19, fibroblast growth factor 19; FGF23, fibroblast growth factor 23; CCL7, C-C motif chemokine ligand 7; TNFSF14, TNF superfamily member 14.

The primary outcomes for the causal effects of genetically predicted circulating inflammatory proteins on T1D with renal complications were depicted in **Figures 2** and **3**. The MR estimates demonstrated that the genetically elevated predicted levels of IL12B (IVW: OR 1.691, 95% CI 1.179–2.427, P =4.34×10^-3^) and LIF interleukin 6 family cytokine (LIF) (IVW: OR 1.349, 95% CI 1.010–1.801, P =4.23×10^-2^) were associated with an increased risk of T1D with renal complications. Conversely, artemin (ARTN) (IVW: OR 0.702, 95% CI 0.529–0.933, P =1.46×10^-2^), C-C motif chemokine ligand 28 (CCL28) (IVW: OR 0.740, 95% CI 0.549–0.999, P =4.95×10^-2^) and S100 calcium binding protein A12 (S100A12) (IVW: OR 0.729, 95% CI 0.564–0.944, P =1.64×10^-2^) were associated with reduced risks (**Table 1**; **Figure 4**). Cochran’s Q test indicated heterogeneity for IL-12B (IVW-derived Q statistic = 38.763; P = 0.007), and the MR-PRESSO test identified an outlier, the overall estimates were generally consistent after correction. The MR-Egger intercept analysis indicated no pleiotropy for IL-12B (P > 0.05). For LIF, ARTN, CCL28, and S100A12, neither MR-Egger intercept analysis (P > 0.05) nor Cochran’s Q test (P > 0.05) indicated the presence of pleiotropy or heterogeneity, and no outliers were detected by MR-PRESSO (**Supplementary Figure 1**).

**Table 1:** MR and colocalization analyses results of causal associations between circulating inflammatory proteins and diabetic renal complications.

outcome	exposure	nSNP	IVW		Cochran’s Q test		MR-Egger intercept analysis	Colocalization analysis
			OR (95%CI)	P value	IVW derived Q statistic	IVW derived P value	P value	PH4
T1D with renal complications								
	**ARTN**	19	0.702 (0.529–0.933)	1.46E-02	23.745	0.164	0.186	0.996
	**CCL28**	21	0.740 (0.549–0.999)	4.95E-02	22.887	0.294	0.620	0.903
	**S100A12**	16	0.729 (0.564–0.944)	1.64E-02	9.053	0.875	0.214	0.997
	**IL-12B**	21	1.691 (1.179–2.427)	4.34E-03	38.763	0.007	0.469	0.943
	**LIF**	16	1.349 (1.010–1.801)	4.23E-02	13.578	0.558	0.761	0.973
T2D with renal complications								
	**DNER**	17	0.701 (0.527–0.932)	1.45E-02	25.048	0.069	0.320	0.347
	**IL-13**	17	0.792 (0.644–0.974)	2.72E-02	13.565	0.631	0.703	0.984
	**FGF19**	20	1.202 (1.009–1.432)	3.93E-02	15.437	0.694	0.427	0.979
	**FGF23**	15	1.379 (1.035–1.837)	2.82E-02	18.527	0.184	0.920	0.954
	**CCL7**	16	1.385 (1.111–1.725)	3.76E-03	17.743	0.276	0.897	0.991
	**TNFSF14**	20	1.244 (1.066–1.451)	5.63E-03	7.570	0.991	0.714	0.976

MR, mendelian randomization; SNP, single nucleotide polymorphism; IVW, inverse-variance weighted; PP, posterior probability; T1D, type 1 diabetes; T2D, type 2 diabetes; ARTN, artemin; CCL28, C-C motif chemokine ligand 28; S100A12, S100 calcium binding protein A12; IL-12B, interleukin 12B; LIF, LIF interleukin 6 family cytokine; DNER, delta/notch like EGF repeat containing; IL-13, interleukin 13; FGF19, fibroblast growth factor 19; FGF23, fibroblast growth factor 23; CCL7, C-C motif chemokine ligand 7; TNFSF14, TNF superfamily member 14.

### Causal estimates of circulating inflammatory proteins on T2D with renal complications

**Figure 5:**
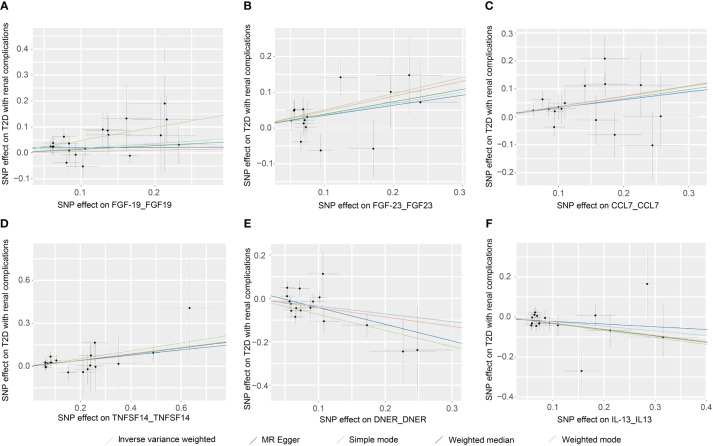
Scatter plots of causal associations for genetically predicted levels of FGF19 **(A)**, FGF23 **(B)**, CCL7 **(C)**, TNFSF14 **(D)**, DNER **(E)**, and IL-13 **(F)** on T2D with renal complications used IVW, MR-Egger, weighted median, weighted mode, and simple mode methods in MR analysis. MR, mendelian randomization; IVW, inverse-variance weighted; T2D, type 2 diabetes; FGF19, fibroblast growth factor 19; FGF23, fibroblast growth factor 23; CCL7, C-C motif chemokine ligand 7; TNFSF14, TNF superfamily member 14; DNER, delta/notch like EGF repeat containing; IL-13, interleukin 13.

The principal results for the causal effects of genetically predicted circulating inflammatory proteins on T2D with renal complications were showed in **Figures 2** and **5**. The MR estimates indicated that elevated levels of fibroblast growth factor 19 (FGF19) (IVW: OR 1.202, 95% CI 1.009–1.432, P =3.93×10^-2^), fibroblast growth factor 23 (FGF23) (IVW: OR 1.379, 95% CI 1.035–1.837, P =2.82×10^-2^), C-C motif chemokine ligand 7 (CCL7) (IVW: OR 1.385, 95% CI 1.111–1.725, P =3.76×10^-3^) and TNF superfamily member 14 (TNFSF14) (IVW: OR 1.244, 95% CI 1.066–1.451, P =5.63×10^-3^) were potential risk factors of T2D with renal complications, whereas elevated levels of delta/notch like EGF repeat containing (DNER) (IVW: OR 0.701, 95% CI 0.527–0.932, P =1.45× 10^-2^) and IL-13 (IVW: OR 0.792, 95% CI 0.644–0.974, P =2.72× 10^-2^) may serve as protective factors (**Table 1**; **Figure 4**). For FGF19, FGF23, CCL7, TNFSF14, DNER and IL-13, no significant heterogeneity was observed by Cochran’s Q test (P > 0.05), and MR-PRESSO identified no outliers. The MR-Egger intercept analysis suggested no pleiotropy (P > 0.05) (**Supplementary Figure 2**).

### Colocalization analysis

Through colocalization analysis, we can provide robust evidence supporting the causal relationship between proteins identified via MR analysis and diabetes with renal complications. This method minimizes confounding effects from genetic backgrounds, thus enhancing the reliability of causal inference. The primary objectives of colocalization analysis are to identify shared causal variants (common SNPs) and determine whether a variant affects both protein levels and disease risk, indicating a potential causal relationship.

Colocalization analysis was performed for proteins that were significantly related to diabetic renal complications in MR analysis. Our colocalization analysis revealed that H4 PPs between ARTN, CCL28, S100A12, IL-12B, and LIF levels and T1D with renal complications were greater than 0.8, respectively (ARTN at H4 = 0.996, CCL28 at H4 = 0.903, S100A12 at H4 = 0.997, IL-12B at H4 = 0.943, and LIF at H4 = 0.973) (**Supplementary Figure 3**). Similar findings were observed for proteins in T2D with renal complications (IL-13 at H4 = 0.984, FGF19 at H4 = 0.979, FGF23 at H4 = 0.954, CCL7 at H4 = 0.991, and TNFSF14 at H4 = 0.976) (**Supplementary Figures 4B–F**). It was highly supported that these two traits shared single causal variant within the 1Mb region of the gene, reinforcing the genetic and biological connection between these proteins and diabetic renal complications. However, we found that the H4 PP of DNER levels and T2D with renal complications was low (H4 = 0.347), and as such indicated weak colocalization evidence in support of a shared causal variant (**Supplementary Figure 4A**). These finding might provide valuable insights into the genetic mechanisms and potential therapeutic targets for diabetic renal complications, underscoring the importance of specific proteins in disease progression and treatment strategies.

## Discussion

We performed proteome-wide MR and colocalization analyses to investigate the causal effects of 91 circulating inflammatory proteins on diabetic renal complications. Our findings demonstrated that genetically predicted higher levels of IL-12B and LIF likely increased the risk of T1D with renal complications, whereas higher levels of ARTN, CCL28, and S100A12 were related to a decreased risk. Likewise, elevated levels of FGF19, FGF23, CCL7 and TNFSF14 were potential risk factors of T2D with renal complications, whereas elevated levels of DNER and IL-13 may serve as protective factors. Colocalization evidence reinforced these associations, suggesting that in addition to DNER, the other circulating inflammatory proteins showed evidence of sharing causal SNPs with diabetic renal complications. Prior studies have shown that proteins including S100A12, IL-12B, LIF, FGF19, FGF23, CCL7, TNFSF14, and IL-13 play a role in the inflammatory processes of diabetic renal complications. However, direct evidence elucidating how ARTN, CCL28, and DNER influence diabetic renal complications and their potential as therapeutic targets remains scarce.

The IL-12B gene encodes the p40 subunit of cytokines IL-12 and IL-23, which are pivotal in modulating immune responses through the stimulation of Th1 and Th17 cell differentiation and activation (24). T1D predominantly arises from autoimmune reactions that destroy pancreatic beta cells, a process that IL-12 and IL-23 might intensify in autoimmune conditions (25). Elevated levels of IL-12 and IL-23 can enhance Th1 and Th17 cell-mediated inflammation, culminating in an excessive production of pro-inflammatory cytokines, including interferon gamma (IFN-γ), TNF-α, IL-17, and IL-22 (25, 26). Such cytokine overproduction likely initiates inflammation and subsequent tissue damage in the kidneys. LIF, a member of the interleukin 6 cytokine family, exhibits multifunctional properties. Xu et al. (27) found that LIF was significantly upregulated in renal fibrotic lesions in both humans and mice, with its mRNA expression inversely associated with eGFR. Meanwhile, elevated LIF levels were observed in individuals with DN. Mechanistically, LIF may contribute to the activation of renal mesangial cells under hyperglycemic conditions by activating the STAT and MAPK signaling pathways (28, 29). Additionally, LIF has been implicated in the upregulation of MCP-1 expression in glomerular mesangial cells, thereby fostering glomerular inflammation (30).

Studies have reported that T2D patients with increased plasma FGF23 concentrations experienced a higher all-cause mortality rate (31, 32). FGF23 was identified as a significantly prognostic marker for renal outcomes, particularly in DN patients with severe proteinuria (33). Inhibition of FGF23 in db/db mice has been shown to attenuate inflammation and fibrosis, thereby ameliorating outcomes of DN (34). Moreover, plasma levels of FGF23 tend to escalate during the initial phases of CKD with renal function declining, a response that appear to enhance phosphate excretion per nephron, maintaining phosphate homeostasis. However, studies have suggested that the severity of tubular damage and interstitial fibrosis correlated positively with the phosphate excretion per nephron unit (35).

Upon release into damaged or infected tissue, CCL7 facilitates the recruitment of macrophages and monocytes to inflammatory sites by interacting with CC chemokine receptor 2 (CCR2). This process can potentially intensify inflammatory responses and contribute to disease progression (36). Studies have indicated that serum CCL7 levels were elevated in patients with T2D. It is posited that CCL7 may play a role in the onset of adipose tissue inflammation and insulin resistance in T2D and could be linked to the advancement of inflammation and fibrosis in DN (37). TNFSF14 is known for its pro-inflammatory properties. Preliminary research has shown that TNFSF14 concentrations were higher in individuals with T2D and that it can induce islet cell dysfunction *in vitro (* 38). Evidence suggested that the absence of TNFSF14 can enhance glucose tolerance and insulin sensitivity, alter immune cell phenotypes, and decrease the secretion of inflammatory cytokines, including IL-6, IL-8, IL-17, and TNF-α (39).

IL-13 suppresses Th1 and Th17 cell-mediated immune responses, augments Th2 cell responses, and prompts macrophage activation to reduce pro-inflammatory cytokine production (such as IL-1β, TNF-α, IL-17) while fostering the generation of anti-inflammatory cytokines (such as IL-10) (40). This cascade of events manifests as an anti-inflammatory effect. Furthermore, IL-13 was posited to be pivotal in ameliorating kidney fibrosis (41). Evidence indicates that IL-13 modulates glucose homeostasis via the IL-13rα1-STAT3 signaling pathway in hepatocytes, potentially providing a therapeutic target for glucose regulation in T2D (42). While IL-13 could decelerate T2D progression, particularly when renal complications are present, its precise biological roles and prospective therapeutic targets deserve additional exploration.

The proteins mentioned above have been corroborated by prior research. Through MR and colocalization analyses, we have further reinforced the causal links between these proteins and diabetic renal complications. Simultaneously, we have identified several unverified proteins that may offer novel insights into the pathogenic mechanisms underlying diabetic renal complications.

ARTN predominantly functions within the nervous system, playing a crucial role in the promotion of neuronal growth and development (43). CCL28 falls under the CC chemokine family classification. By interacting with specific receptors, such as CCR10, it orchestrates the migration of immune cells and is involved in inflammatory processes and immune regulation (44). Our MR estimates indicated that ARTN and CCL28 may confer a protective effect against T1D with renal complications, although this has not been demonstrated in extant studies. In the context of diabetic nephropathy, we postulated that ARTN may be instrumental in supporting the survival and repair of injured neurons or other cell types, while CCL28 is likely implicated in modulating inflammation, immune responses, and tissue repair mechanisms. The implications of these novel findings warrant further investigation. Besides, our findings also indicated an inverse correlation between increased DNER levels and the risk of T2D with renal complications. However, this association lacked evidence from colocalization analysis.

Studies have demonstrated that serum S100A12 levels were elevated in T1D patients, and such elevations were associated with inflammatory responses and diabetes in patients with stage 5 chronic kidney disease (CKD) (45, 46). Contrary to these findings, our results suggested that S100A12 may act as a protective protein in T1D with renal complications. Prior research has identified FGF19 as a potential target for managing diabetes and its associated complications (47). It is also contradicted with our MR results. This suggests a pleiotropic role for inflammatory proteins inside diabetic renal complications that warrants further investigation.

For future research, it is crucial to investigate the biological pathways and functional roles of these proteins in diabetic renal complications. Employing advanced techniques such as single-cell RNA sequencing, proteomics, and gene editing in both cellular and animal models will be essential. Moreover, expanding studies to include diverse population cohorts will enhance the generalizability of the findings. Importantly, the genes corresponding to these proteins—S100A12, IL12B, LIF, FGF19, FGF23, CCL7, TNFSF14, IL-13, ARTN, and CCL28—are considered druggable, with their tier levels being Tier 3B, Tier 1, Tier 3A, Tier 3B, Tier 3B, Tier 3A, Tier 1, Tier 1, Tier 3B, and Tier 3B, respectively (48). Investigating the therapeutic potential of targeting these proteins could lead to personalized treatment strategies, ultimately improving clinical outcomes for patients with diabetic renal complications. Lastly, to facilitate clinical translation, future research should incorporate preclinical studies to evaluate the efficacy and safety of potential therapies targeting these proteins, followed by clinical trials to assess their effectiveness in human subjects. Additionally, establishing reliable biomarkers for early detection and treatment monitoring will be essential.

There are some limitations in our study. Firstly, a limited number of SNPs reached the significance threshold of 5×10^-8^ for the MR analysis. Therefore, with reference to similar studies, we adopted a relatively lenient genetic instrument threshold of 1×10^-5^ (19). Besides, our analyses focused on populations of European descent, reducing demographic bias but potentially limiting the generalizability of our findings to other ethnic groups. Future studies should include diverse population cohorts from various ethnic backgrounds. In addition, the p values for the causal associations we observed reached the thresholds of suggestive association rather than the Bonferroni-corrected thresholds. Thus, we cannot rule out the possibility of false positives in the observed associations between inflammatory proteins and diabetic renal complications. Lastly, horizontal pleiotropy is also a potential constraint in MR studies. We further verified our findings through sensitivity analyses using methods like MR-PRESSO, weighted median, MR-Egger, weighted mode and simple mode to mitigate pleiotropy, reinforcing the reliability and robustness of our findings. Although these tests provided no evidence of horizontal pleiotropy, residual pleiotropy is impossible to completely exclude.

## Conclusions

In conclusion, our two-sample MR analysis provided evidence for the causal relationships between circulating inflammatory proteins and diabetic renal complications. Previous research has established that parts of these proteins play roles in the progression of diabetic renal complications. It was notable that IL12B, LIF and S100A12 emerged as potential therapeutic targets for T1D with renal complications, and that FGF19, FGF23, CCL7, TNFSF14 and IL-13 also deserved attention for T2D with renal complications. Future research should be directed towards elucidating the mechanisms of action and signaling pathways of these prospective targets, thus aiding in the development of novel therapeutic methods for diabetic renal complications.

## Data availability statement

The original contributions presented in the study are included in the article/**Supplementary Material**. Further inquiries can be directed to the corresponding author.

## Author contributions

WL: Data curation, Formal analysis, Writing – original draft, Visualization. JZ: Data curation, Formal analysis, Writing – original draft. DZ: Writing – review & editing. LZ: Conceptualization, Funding acquisition, Writing – review & editing.

## Supplementary material

Supplementary Figure 1 Leave one out analysis of causal associations for genetically predicted levels of IL-12B **(A)**, LIF **(B)**, ARTN **(C)**, CCL28 **(D)**, and S100A12 **(E)** in T1D with renal complications. T1D, type 1 diabetes; IL-12B, interleukin 12B; LIF, LIF interleukin 6 family cytokine; ARTN, artemin; CCL28, C-C motif chemokine ligand 28; S100A12, S100 calcium binding protein A12.

Supplementary Figure 2 Leave one out analysis of causal associations for genetically predicted levels of FGF19 **(A)**, FGF23 **(B)**, CCL7 **(C)**, TNFSF14 **(D)**, DNER **(E)**, and IL-13 **(F)** in T2D with renal complications. T2D, type 2 diabetes; FGF19, fibroblast growth factor 19; FGF23, fibroblast growth factor 23; CCL7, C-C motif chemokine ligand 7; TNFSF14, TNF superfamily member 14; DNER, delta/notch like EGF repeat containing; IL-13, interleukin 13.

Supplementary Figure 3 Colocalization analysis was performed for identified proteins that were significantly related to T1D with renal complications in MR analysis. **(A)** Colocalization analysis of the cis-pQTL for ARTN **(B)** Colocalization analysis of the cis-pQTL for CCL28 **(C)** Colocalization analysis of the cis-pQTL for S100A12 **(D)** Colocalization analysis of the cis-pQTL for IL-12B **(E)** Colocalization analysis of the cis-pQTL for LIF. MR, mendelian randomization; T1D, type 1 diabetes; pQTL, protein quantitative trait loci; IL-12B, interleukin 12B; LIF, LIF interleukin 6 family cytokine; ARTN, artemin; CCL28, C-C motif chemokine ligand 28; S100A12, S100 calcium binding protein A12.

Supplementary Figure 4 Colocalization analysis was performed for identified proteins that were significantly related to T2D with renal complications in MR analysis. **(A)** Colocalization analysis of the cis-pQTL for DNER **(B)** Colocalization analysis of the cis-pQTL for IL-13 **(C)** Colocalization analysis of the cis-pQTL for FGF19 **(D)** Colocalization analysis of the cis-pQTL for FGF23 **(E)** Colocalization analysis of the cis-pQTL for CCL7 **(F)** Colocalization analysis of the cis-pQTL for TNFSF14. MR, mendelian randomization; T2D, type 2 diabetes; pQTL, protein quantitative trait loci; FGF19, fibroblast growth factor 19; FGF23, fibroblast growth factor 23; CCL7, C-C motif chemokine ligand 7; TNFSF14, TNF superfamily member 14; DNER, delta/notch like EGF repeat containing; IL-13, interleukin 13.
