# Supplementary figures and images for: Role of circulating inflammatory protein in the development of diabetic renal complications: proteome-wide Mendelian randomization and colocalization analyses
Source: Front Endocrinol (Lausanne). 2024 Jul 8;15:1406442. doi: 10.3389/fendo.2024.1406442 (PMC11260607; doi:10.3389/fendo.2024.1406442)

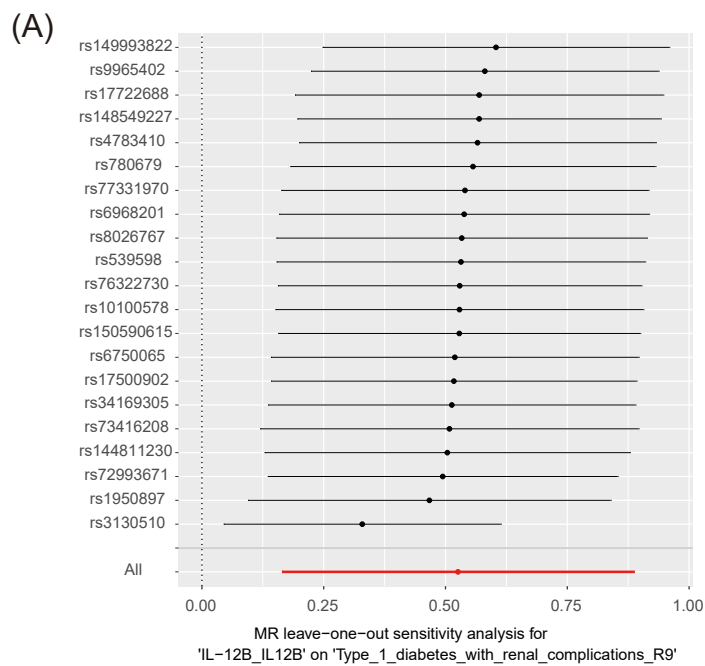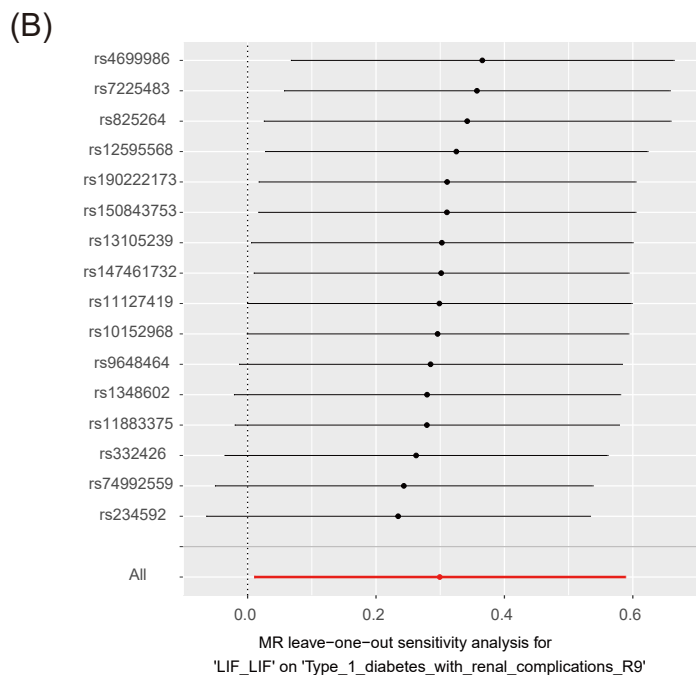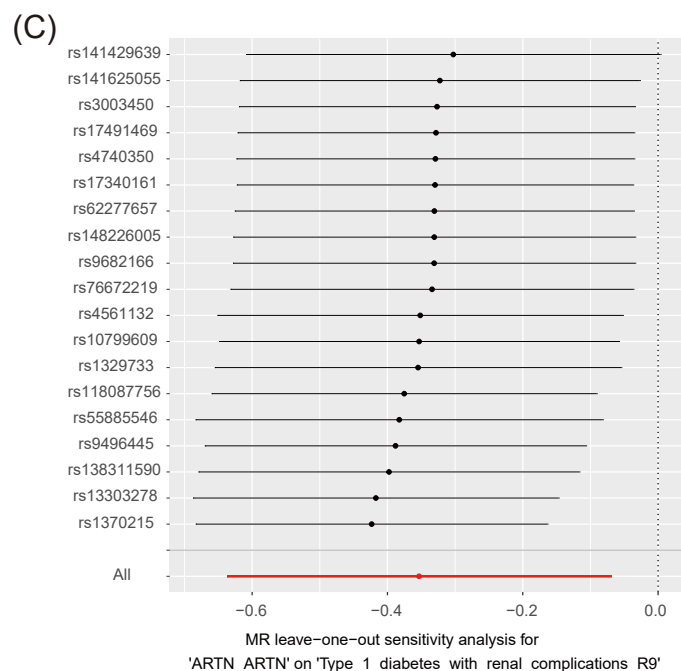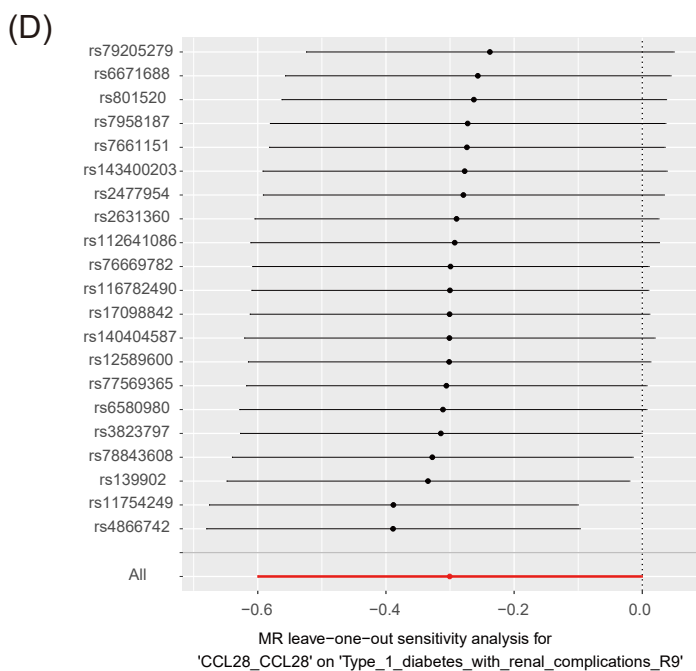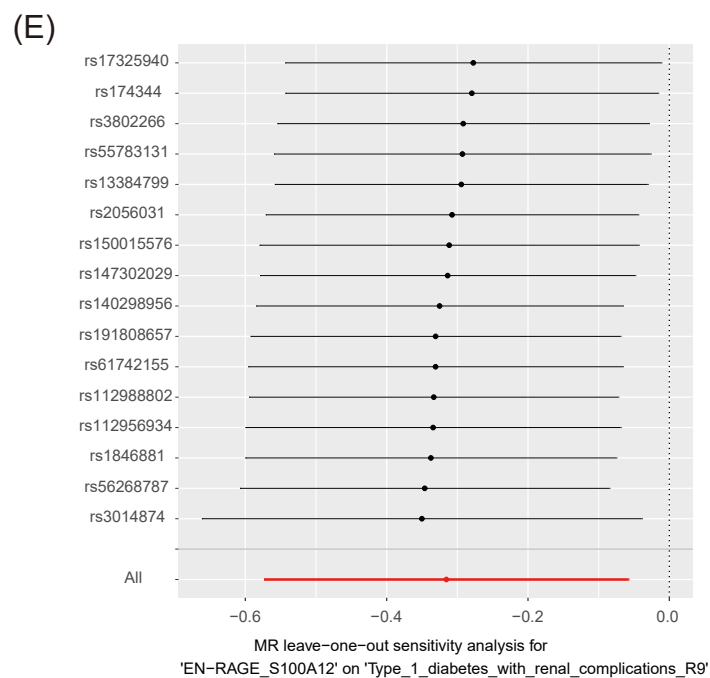

Supplement: Supplementary Figure 1 — Leave one out analysis of causal associations for genetically predicted levels of IL-12B (A), LIF (B), ARTN (C), CCL28 (D), and S100A12 (E) in T1D with renal complications. T1D, type 1 diabetes; IL-12B, interleukin 12B; LIF, LIF interleukin 6 family cytokine; ARTN, artemin; CCL28, C-C motif chemokine ligand 28; S100A12, S100 calcium binding protein A12. [file DataSheet_1.pdf]

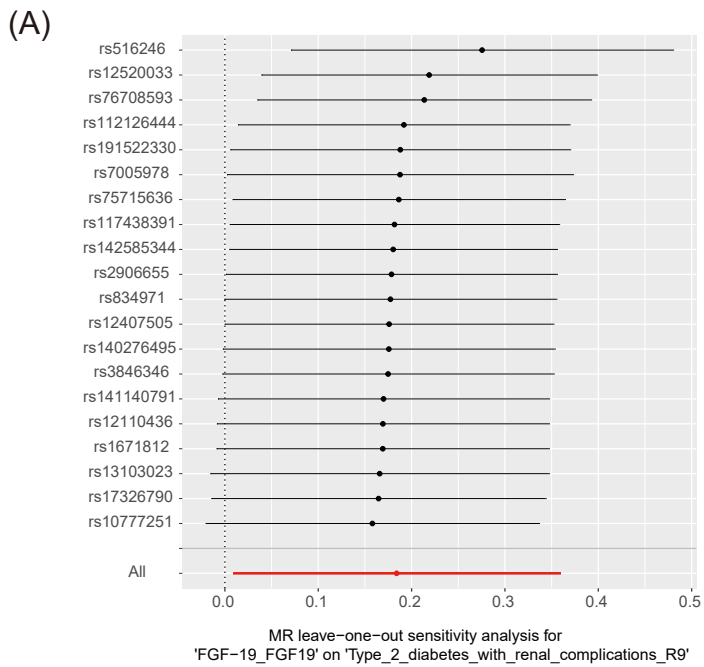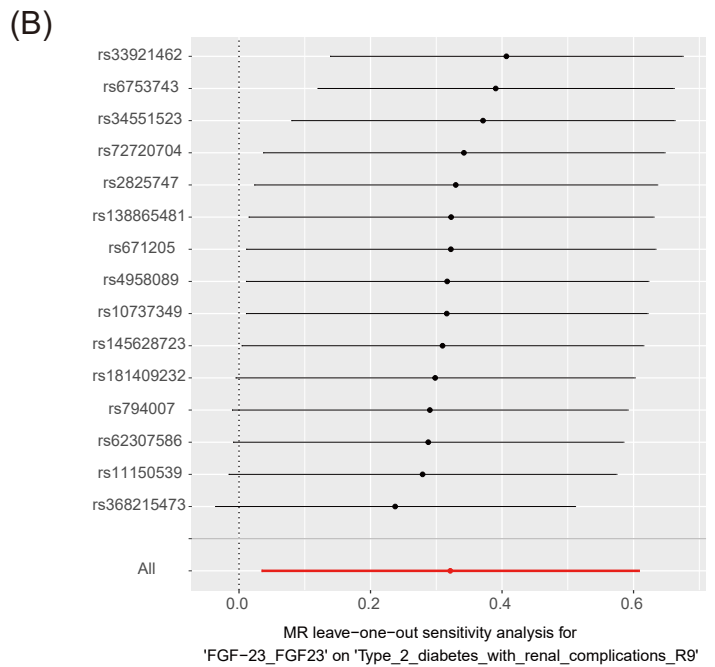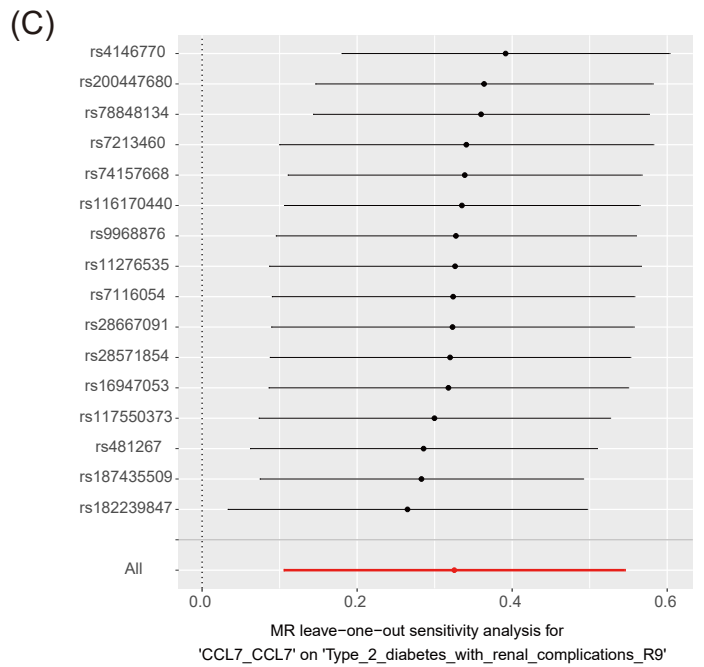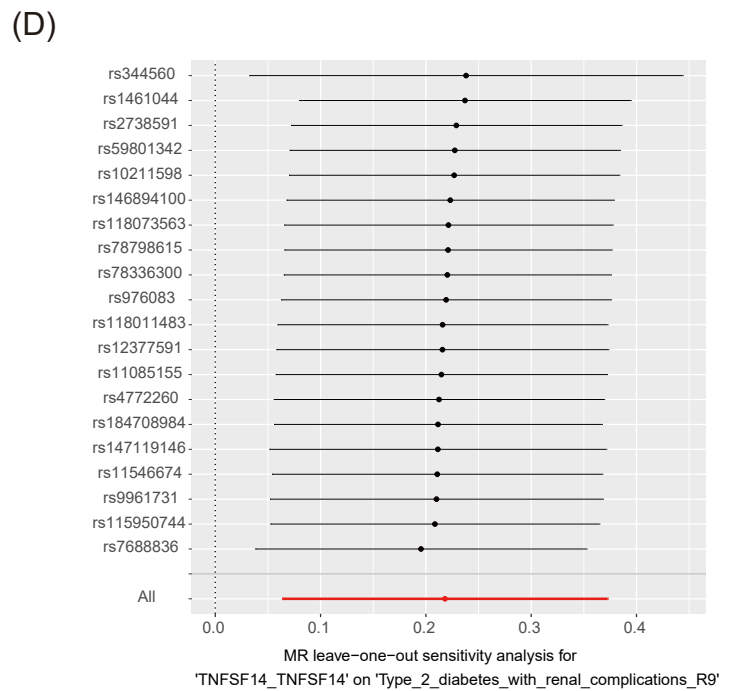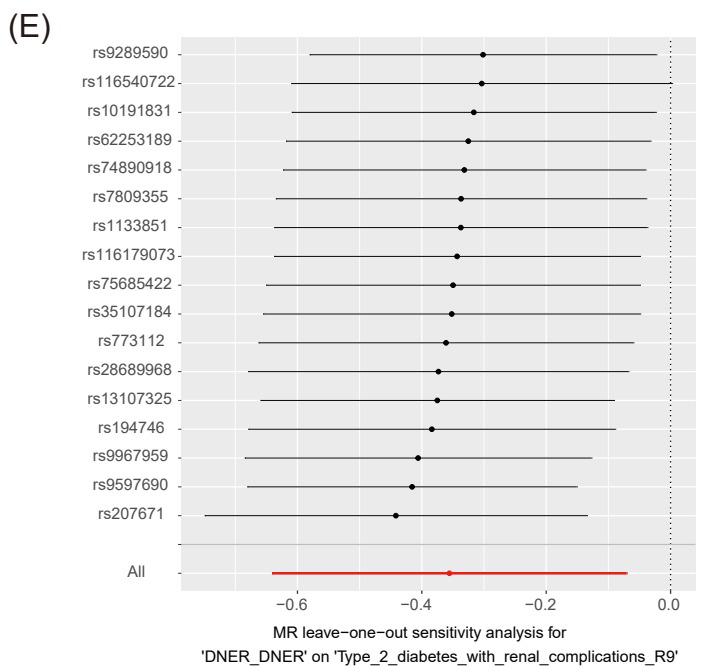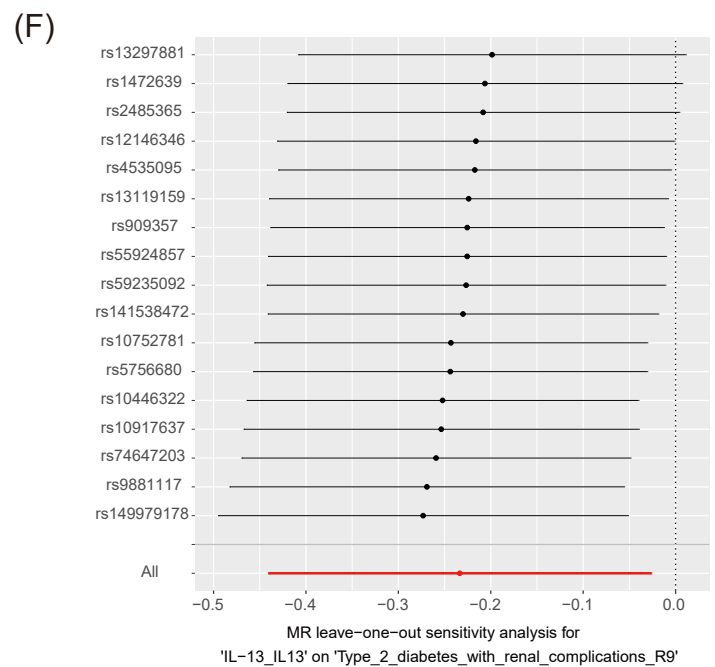

Supplement: Supplementary Figure 2 — Leave one out analysis of causal associations for genetically predicted levels of FGF19 (A), FGF23 (B), CCL7 (C), TNFSF14 (D), DNER (E), and IL-13 (F) in T2D with renal complications. T2D, type 2 diabetes; FGF19, fibroblast growth factor 19; FGF23, fibroblast growth factor 23; CCL7, C-C motif chemokine ligand 7; TNFSF14, TNF superfamily member 14; DNER, delta/notch like EGF repeat containing; IL-13, interleukin 13. [file DataSheet_2.pdf]

(A)

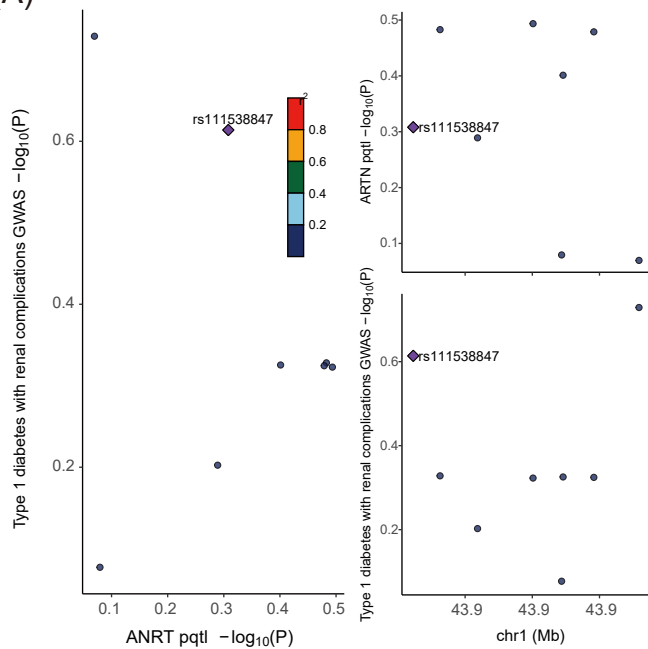

(B)

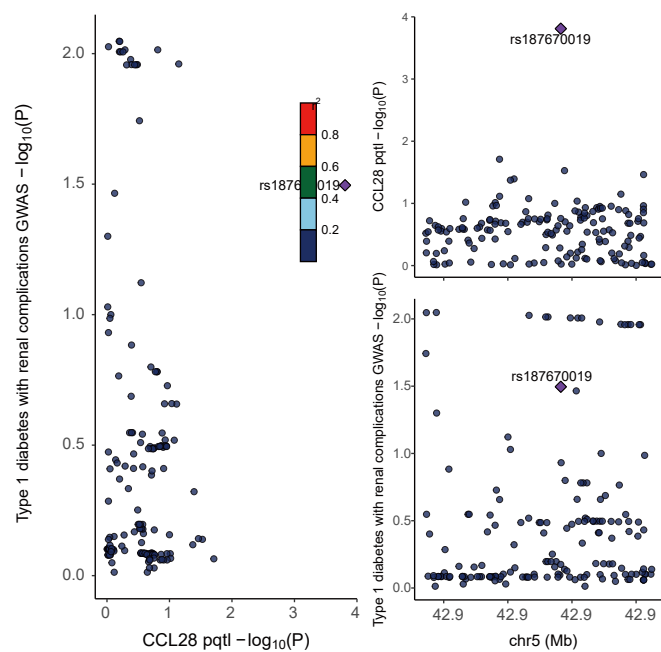

(C)

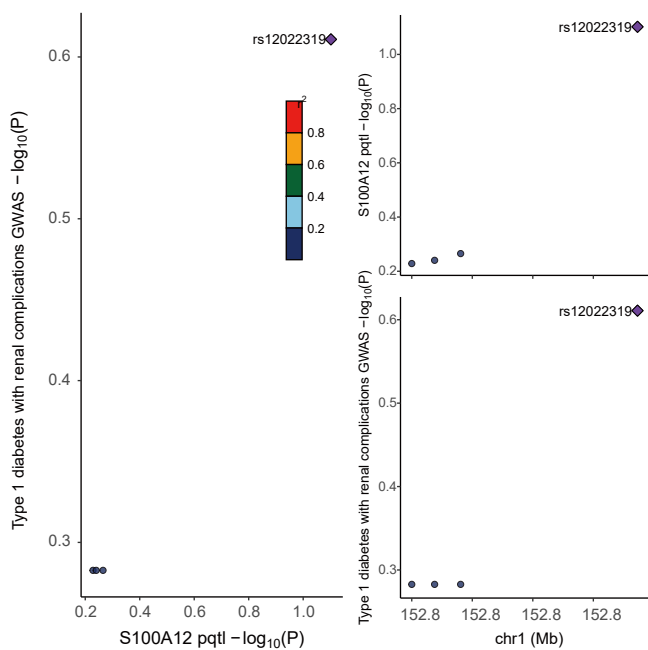

(D)

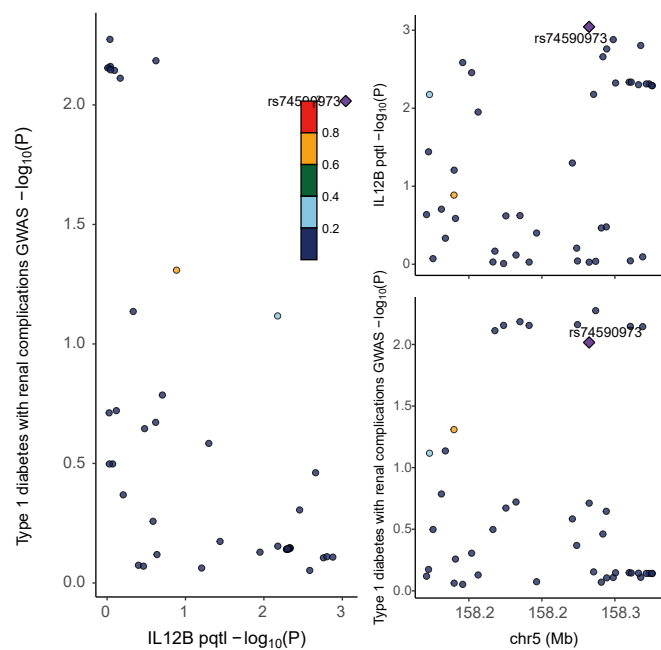

(E)

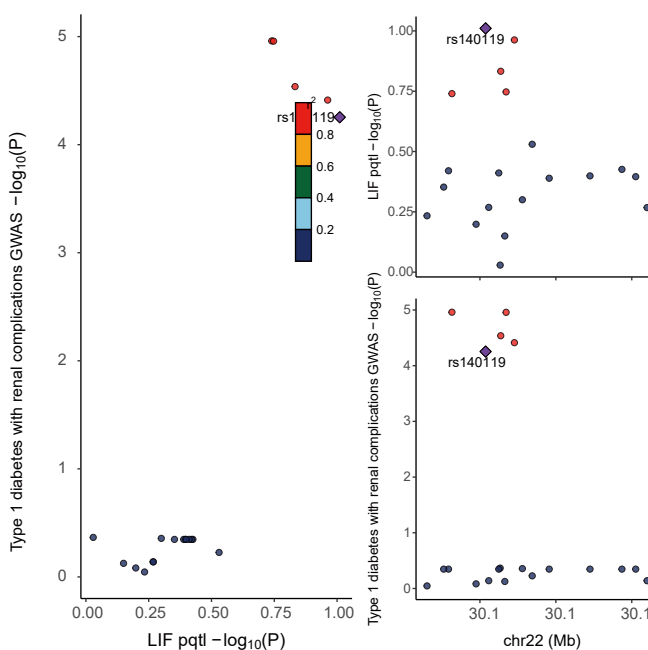

Supplement: Supplementary Figure 3 — Colocalization analysis was performed for identified proteins that were significantly related to T1D with renal complications in MR analysis. (A) Colocalization analysis of the cis-pQTL for ARTN (B) Colocalization analysis of the cis-pQTL for CCL28 (C) Colocalization analysis of the cis-pQTL for S100A12 (D) Colocalization analysis of the cis-pQTL for IL-12B (E) Colocalization analysis of the cis-pQTL for LIF. MR, mendelian randomization; T1D, type 1 diabetes; pQTL, protein quantitative trait loci; IL-12B, interleukin 12B; LIF, LIF interleukin 6 family cytokine; ARTN, artemin; CCL28, C-C motif chemokine ligand 28; S100A12, S100 calcium binding protein A12. [file DataSheet_3.pdf]

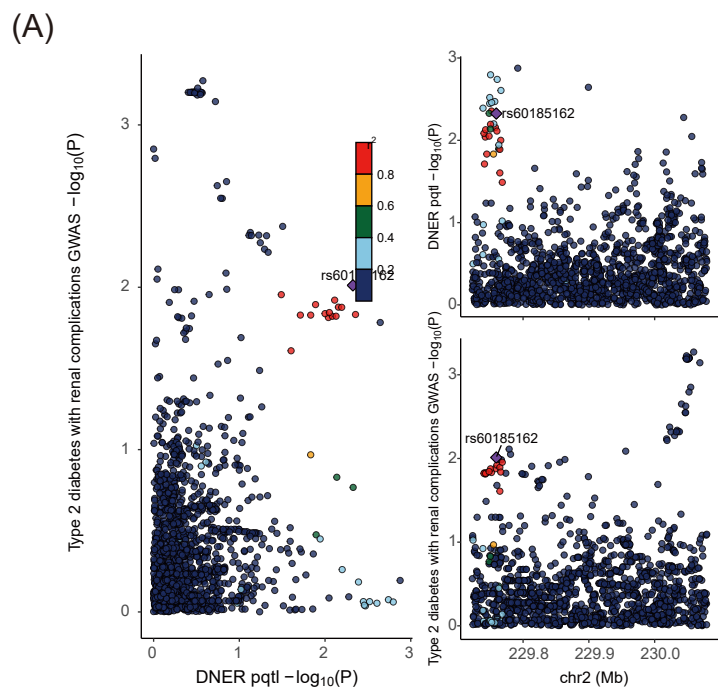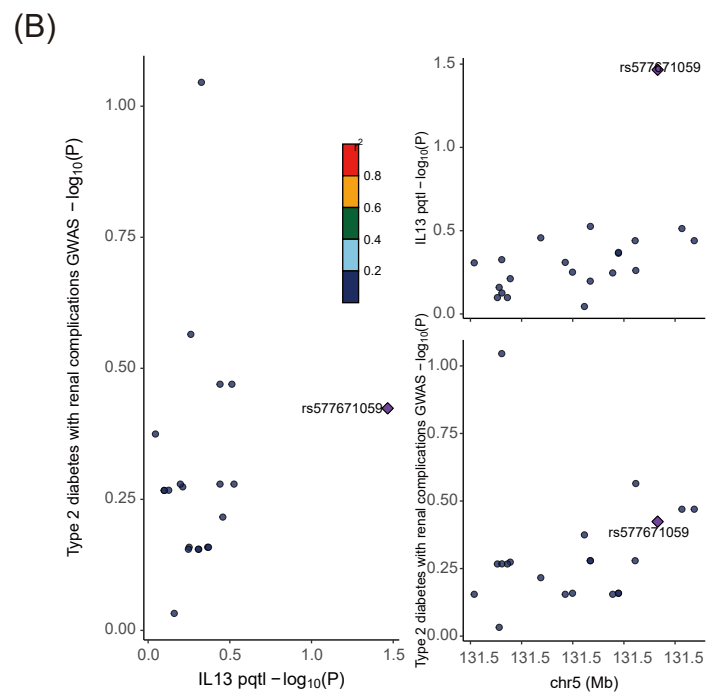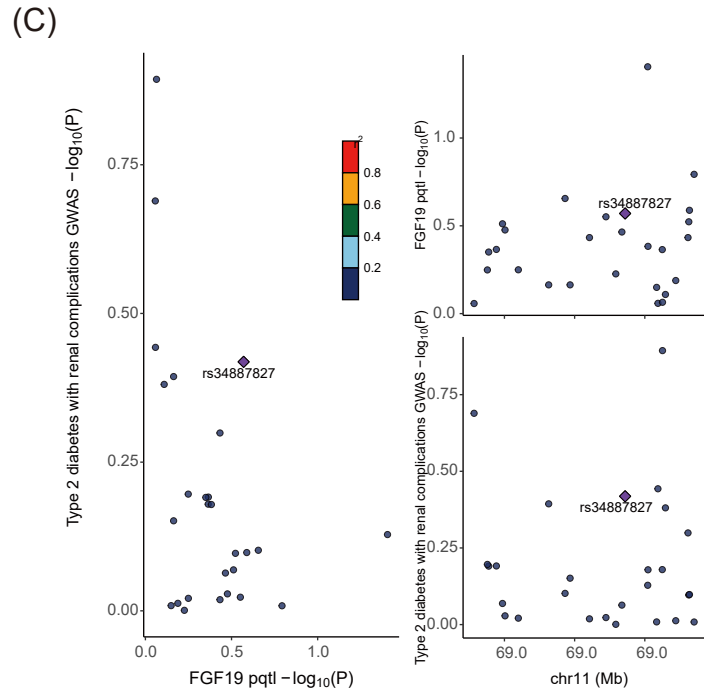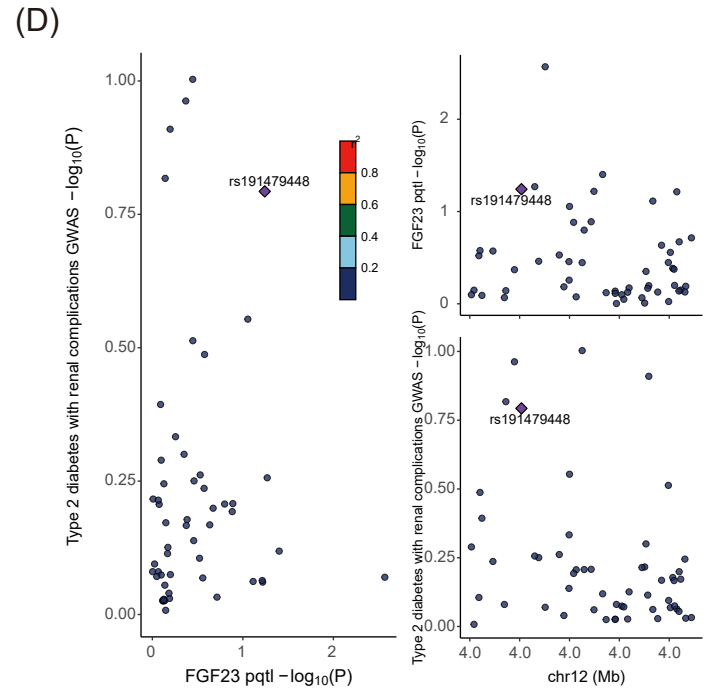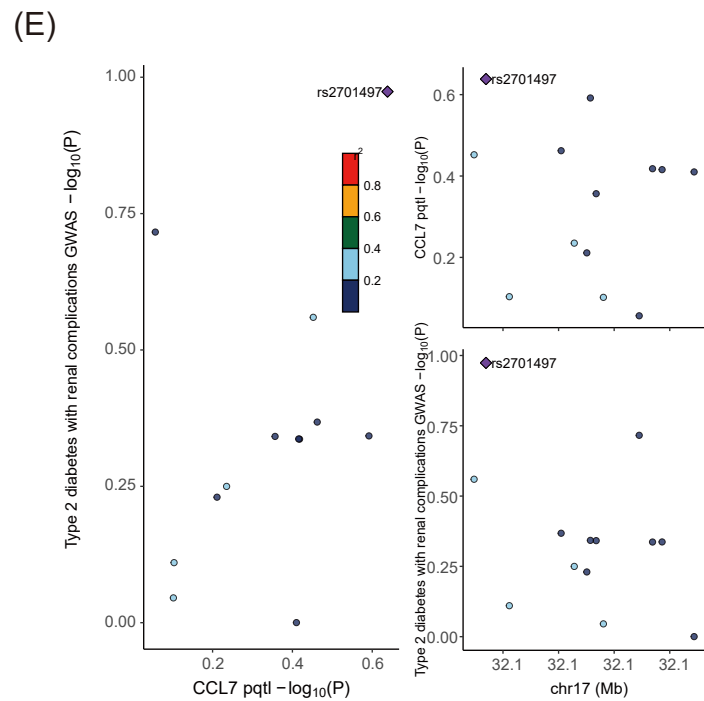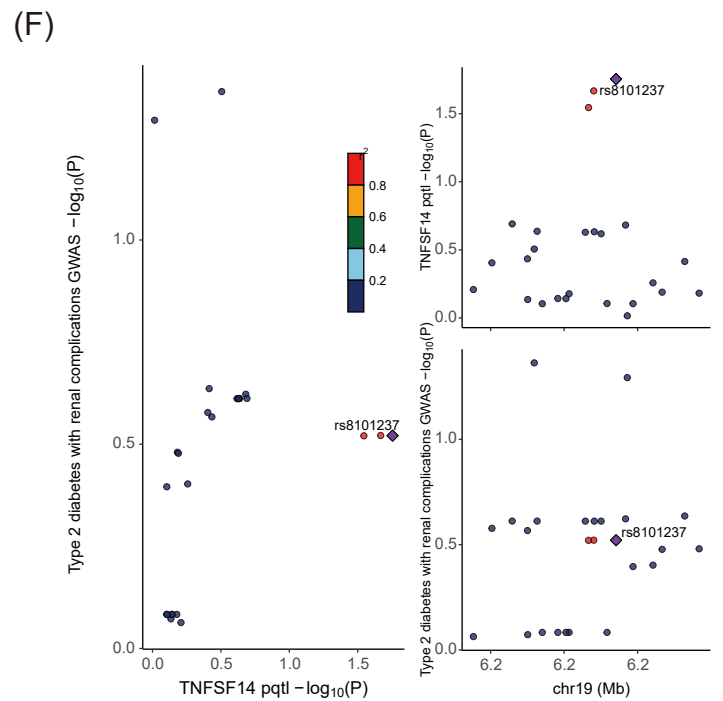

Supplement: Supplementary Figure 4 — Colocalization analysis was performed for identified proteins that were significantly related to T2D with renal complications in MR analysis. (A) Colocalization analysis of the cis-pQTL for DNER (B) Colocalization analysis of the cis-pQTL for IL-13 (C) Colocalization analysis of the cis-pQTL for FGF19 (D) Colocalization analysis of the cis-pQTL for FGF23 (E) Colocalization analysis of the cis-pQTL for CCL7 (F) Colocalization analysis of the cis-pQTL for TNFSF14. MR, mendelian randomization; T2D, type 2 diabetes; pQTL, protein quantitative trait loci; FGF19, fibroblast growth factor 19; FGF23, fibroblast growth factor 23; CCL7, C-C motif chemokine ligand 7; TNFSF14, TNF superfamily member 14; DNER, delta/notch like EGF repeat containing; IL-13, interleukin 13. [file DataSheet_4.pdf]
